# Morphological, physiological and biochemical response of L*allemantia* species to elevated temperature and light duration during seed development

**DOI:** 10.1016/j.heliyon.2023.e15149

**Published:** 2023-04-11

**Authors:** Arezoo Paravar, Saeideh Maleki Farahani, Alireza Rezazadeh

**Affiliations:** a Department of Crop Production and Plant Breeding, College of Agriculture, Shahed University, Tehran, Iran; b Department of Plant Protection, College of Agriculture, Shahed University, Tehran, Iran

**Keywords:** Maternal environment, Seed quality, Hydrogen peroxide, Lipid peroxidation, Fatty acids

## Abstract

Seed weight, storability, and germinability can depend on maternal plant's environment. However, there is slight information about the effect of light and temperature on seed quality of *Lallemantia* species. The purpose of this research was to determine the properties of physio-biochemical of maternal plant, seed quality, and seed chemical composition of *Lallemantia* species (*Lallemantia iberica* and *Lallemantia royleana*) under temperature (15 °C, 25 °C, and 35 °C) and photoperiod (8 hd^-1^, 16 hd^-1^, and 24 hd^-1^) maternal plants environment. Increasing temperature and photoperiod caused a reduction in leaf chlorophyll, stomatal movement, total soluble sugar, superoxide dismutase (SOD), catalase (CAT), and ascorbate peroxidase (APX) enzymes activities, and an increment in malondialdehyde (MDA) and hydrogen peroxide (H2O2) content of seeds. However, the highest weight, germination, vigor index, and longevity, seed chemical compositions were obtained in offspring which matured under 25 °C for 16 hd^-1^. The highest germination, oil, and relative percentage of fatty acids (oleic acid (OA), linoleic acid (LA), and linolenic acid (LNA)) were obtained in *L. iberica* seeds. On the contrary, longevity, mucilage, and sucrose were more abundant in *L. royleana* seeds. Overall, this research has clearly shown that temperature and light quality and quantity of maternal plant's environment have an immensely effect on producing of seeds with high-quality. However, it is necessary to investigate the impact of the epigenetic mechanisms of the maternal plant on the offspring in future studies.

## Highlights

• Seed quality of *Lallemantia* species was highly influenced by temperature and photoperiod of maternal plant's environment.
• Standard temperature (25 °C) and long day (16 h d^-1^) positively influenced on leaf chlorophyll stomata.
• Increased temperature and photoperiod promoted the accumulation of H_2_O_2_ and MDA in the leaves of maternal plant.
• A correlation between seed longevity, seed mucilage and antioxidant enzyme activities were observed.

## Graphical abstract

**Figure:**
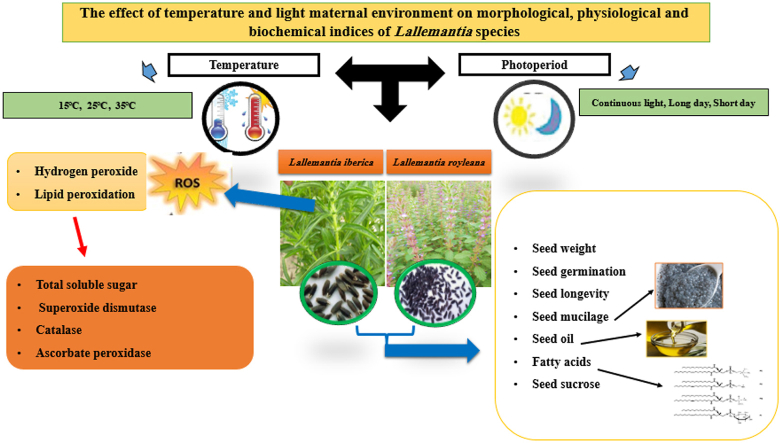

## 1 Introduction

Seed development is a crucial stage in the life cycle of plants and is ecologically and agronomically significant [1,2]. For the successful life cycle of crops in agricultural systems, high-quality seed is crucial to a rise in the production of seeds [3]. In other words, one of agriculture's primary concerns is the production of high-quality seeds [4]. Seed quality includes seed mass, storability, vigor, and germinability, and is used in agricultural systems to show the total value of a seed lot [5]. The main factor contributing to the importance of seed quality is that high-quality seeds have strong seed vigor, which can significantly increase germination rates and seedling establishment [3]. Some useful seed quality indices, such as seed weight, seed composition, germination, and seedling vigor can play a key role in the establishment of plants [6]. Some studies have revealed that there is a positive relationship between seed weight, germination, and seed vigor [4].

Maternal plants' environment can have a determining effect on seed quality, mainly during seed filling [[7], [8], [9]]. It is well known that the various environmental factors can strongly affect the life cycle of mother plants and cause changes in seed quality of offspring [10,11]. The most important environmental factors for seed production are light and temperature which not only cause significant changes in morphological, physiological, and biochemical behaviors in maternal plants but also influence seed weight, seed germination, seed size, seed weight, and seed longevity in offspring [12,13]. The rise in temperature and length of day during seed filling can decrease seed weight due to infertility and seed filling stage reduction [9,14]. Temperature and light in the maternal plant's environment during seed filling may cause a decline in seed oil and seed weight by disrupting the flower's development, pollination, and fertilization [[15], [16], [17]] Researchers reported that unfavorable temperature and light in maternal plants' environments had a detrimental effect on seed weight, germination, vigor, and longevity of *Chenopodium quinoa* Willd [18], *Arabidopsis thaliana* L. [19], *Thlaspi arvense* L [17]. and *Lens culinaris* L [8]. In other words, adverse maternal conditions negatively affect metabolite transport to the sink, resulting in a decline in seed weight, germination, and longevity of offspring [11,20]. A significant downward trend in the weight of seeds matured under high temperature is probably due to the increase in seed growth rate and reduction of the seed filling period [21]. Changes in temperature and length day of maternal plant's environment may influence on seed composition such as carbohydrates and nitrogen [7,22]. Some useful seed quality indices, such as seed weight, seed composition, germination, and vigor, have a key role in the establishment of crops within the farm [6]. Thereby, the growth of maternal plants under a favorable environment of temperature and light can solve a global problem for the seed generation industry, especially with regard to climate change conditions [23].

Some plants can adapt to a specific range of temperatures and photoperiods; however, it may be that unfavorable temperature and light duration cause the induction of oxidative stress and reactive oxygen species (ROS) accumulation in their cellular apoplast [13,24]. ROS accumulation in seeds matured at high temperatures can cause damage to proteins, lipids, and nucleic acids [25]. The mitochondrial destruction under high temperature caused by the accumulated ROS can cause changes in the morphological structures and lipid peroxidation [26]. In some reports, it has been emphasized that owing to physiological deterioration and lipid peroxidation of maternal plant cells, germination and vigor of offspring decrease [4]. Lipid peroxidation can induce the degeneration of seed cells via a ROS attack on essential molecules and the structure of the cell [27]. High temperatures in the maternal plant's environment may cause mitochondrial degeneration and a decrease in adenosine triphosphate accumulation in offspring [28]. The dysfunction of mitochondria and deterioration of the membrane may lead to genetic damage [29,30]. The maternal plant, on the other hand, can protect against induced oxidative stress caused by increases in temperature and length of the maternal environment by expanding enzymatic and non-enzymatic detoxification systems [14,31] The unfavorable light and temperature of maternal plant's environment can induce a reduction in antioxidant enzyme activities, alternation of metabolism, genetic photosynthesis activity of maternal plant [32]. Several studies have found that ROS plays an important role in signal transmission from the chloroplast to the nucleus under adverse light and temperature conditions [33]. Higher ROS can contribute to the decline in seed longevity, germination, and vigor [34].

The *Lallemantia* genus is one of the medicinal plants belonging to the *Lamiaceae* family [35]. Dragon's head (*Lallemantia iberica*) and lady's mantle (*Lallemantia royleana*) are plant species that belong to the Lallemantia sp and are known for their economic benefits [36]. The various compositions of seeds, such as proteins, oil, fatty acids, and carbohydrates, play an important role in the commercial value of seeds [37]. The oil, composition of fatty acids (linolenic acid, linoleic acid, and oleic acid), and mucilage are the main resources in the seeds of *Lallemantia* that are used by the food and pharmaceutical industries [38]. The seed weight, vigor, germination, and longevity, especially the seed composition of L. iberica and L. royleana which develop and mature in various temperatures and light environments, are mostly unclear. As far as we know, the impact of temperature and photoperiod of maternal plant's environment during seed development and maturation on the growth and seed quality of *Lallemantia* species has not been documented. Accordingly, it is necessary to enhance knowledge for the production of quality seeds under different maternal plant's environment. Therefore, thus, this work aims to assess the morphological, physiological, and biochemical response of mother plants and their offspring to elevated temperature and light regime during seed development.

## 2 Material and methods

### 2.1 Plant materials

Seeds of *Lallemantia iberica* and *Lallemantia royleana* (Table 1) were provided in October 2020 from the Agricultural Research Center of Urmia (33°48′ N, 58°44′ E, altitude: 1142 m above sea level). Both species of Lallemantia seeds were stored in a dark, cool room at a stable temperature (10 °C) with a 30% relative humidity until the start of the experiments.

**Table 1:** Characteristics of *Lallemantia* seeds.

Plant species	Seed color	Seed viability (%)	Germination (%)
*Lallemantia iberica*	Light brown	95	98
*Lallemantia royleana*	Dark brown	95	98

### 2.2 Experimental design

A factorial potting experiment was performed on the basis of a completely randomized design with three replicates in nine controlled chambers (BITEC-500, Shimadzu Corp., Kyoto, Japan) of a greenhouse at Shahed University, Tehran, Iran, in November 2020. Both *Lallemantia* species were grown under nine treatments constructed via combinations of temperature (15, 25, and 35 °C) and photoperiod (8, 16, and 24 h d^-1^) (Table 2).

**Table 2:** Details of temperature and photoperiod treatments for *Lallemantia* specie from flowering stage to physiological maturity stage.

	Treatments		Light intensity (μmolm–2s–1)	R:B ratio	Light source
Temperature (°C)	15				
	Low				
	25				
	Standard				
	35	High			
			150	1:8	White fluorescent lamp in color temperature of 4200 K
	8	Long day			
Photoperiod (hd-1)	16	Short day			
	24	Continuous light			

R:B ratio: ratio of red light to blue light.

**Fig. 1:**
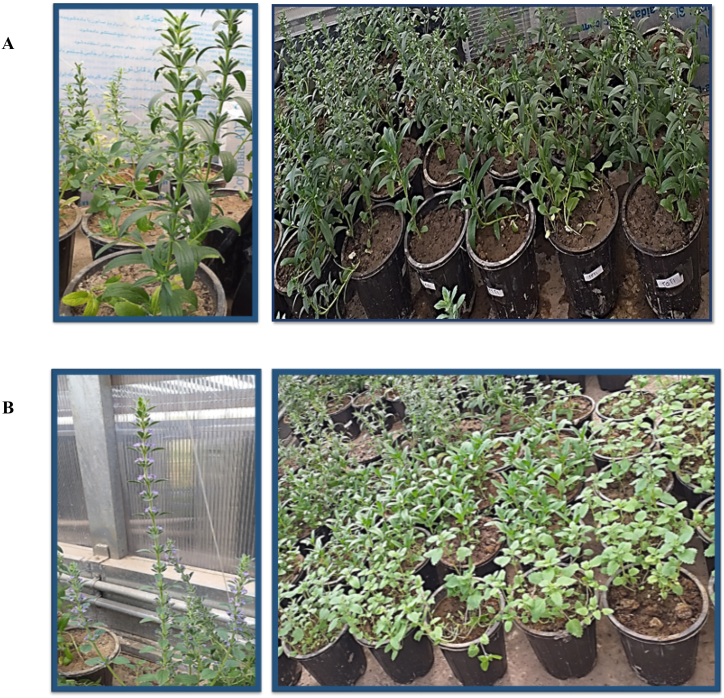
Physiological maturation stage in *in L. iberica* (A) and *L. royleana* (B) in the greenhouse during the experiment.

To do the potting experiment, 54 pots were used, and five seeds were sown in each plastic pot with a 20 cm diameter, which contained a mixture of soil (Table 3) and sand (3:2). From the planting stage to the start of the flowering stage (Fig. 1(A-B)), the plants were grown under white fluorescent lamps (T5-28 W, Shanghai Flower and Biology Lighting Co., China), with a light intensity of 150 μmolm^−2^s^−1^, photoperiod 16 h d^-1^, constant temperature of 23 °C, 70% relative humidity, and an 800 μmolmol^−1^CO_2_ level in controlled chambers. After all the seeds were germinated, only three plants per treatment were selected to grow through the experimental period [20].

**Table 3:** The chemical and physical parameters of potted soil.

Texture	Nitrogen (%)	Iron (mg kg^−1^)	Phosphorus (mg kg^−1^)	Potassium (mg kg^−1^)	Electricity conductivity (dS m^−1^)	pH
Loamy	0.11	2.1	8.52	376	4.2	7.1

To preserve the moisture at 75% of the field capacity, pots were daily weighed, and distilled water was added to each replicate. When most plants had turned yellow (the physiological maturity stage), watering was stopped, and seven days later, the dried plants matured. At the end of the growing season, the seeds of L. iberica (moisture content: 7%) and L. royleana (seed moisture content: 5%) of each maternal plant's environment were harvested at the same time (February 8, 2021). In the following stage, seeds were stored at −80C in screw-cap tubes until the start of trait measurements. The procedures described were carried out for each replication.

### 2.3 Physio-biochemical properties

#### 2.3.1 Leaf chlorophyll

For the measurement of chlorophyll concentration (30 days after flowering), fresh leaves (0.5 g) were powdered using 10 ml acetone (80% v/v). Then, samples were centrifuged (3000 g) for 15 min; then, the absorption of extracts was read at 645 and 663 nm. Chlorophyll *a* and chlorophyll *b* concentration was estimated [39] using equations [1,2]:

[1] Chl a (μg.g^−1^) = 12.7 (A663.6) – 2.69 (A646.6)

[2] Chl b (μg g^−1^) = 22.9 (A646) – 4.68 (A663.6)

#### 2.3.2 Leaf stomata

About 30 days after flowering, three leaves were soaked in glutaraldehyde solution (4% w/v) for 24 h at 12 °C [40]. The stomata of leaves were measured by electron microscopy (CX41, Olympus Corporation, Tokyo, Japan).

#### 2.3.3 Total soluble sugars

To quantify total soluble sugar content, 50 mg fresh leaves (after 30 days flowering) were mixed with sodium phosphate buffer (50 mM, pH 7.5) and centrifuged for 20 min at 12000 rpm [41]. The supernatants were used as the extract of total soluble sugar. Briefly, 0,2 mL of supernatants was added to the anthrone reagent, then the mixture was placed at 80 °C for 40 min. The absorbance was read at 625 nm. Total soluble sugar was calculated using a standard curve of d-glucose [42].

#### 2.3.4 Enzyme extraction and assay of enzyme activities

For the assay of superoxide dismutase (SOD), catalase (CAT), and ascorbate peroxidase (APX), about 30 days after flowering, 200 mg fresh leaves were powdered in an ice-cold condition in 50 mM sodium phosphate buffer (pH 7.0), a-dithiothreitol (2 mM), and EDTA (0.1 mM). The homogenates were centrifuged at 12000 rpm for 15 min at 4 °C. The activity of SOD was assayed using 40 mL enzyme extract, methionine (13 mM), *p*-nitroblue tetrazolium chloride (63 μm), riboflavin (1.5 μm), and 10 μM EDTA. The mixture was incubated for 10 min under florescent light with an intensity 50 μmol m^−2^s^−1^ at 20 min. The reaction was monitored for 3 min at 560 nm. The amount of enzyme was expressed as Units mg^−1^ protein [43]. The CAT reaction mixture included H_2_O_2_ (500 μL 10 mM) and sodium phosphate buffer (1.400 μL 24 mM, pH 7.0). The consumption of H_2_O_2_ was estimated at 240 nm for 3 min after the addition of enzyme extracts to the reaction mixture. CAT activity was estimated using molar extinction coefficient ε = 43.6 M^−1^ cm^−1^ [44]. The reaction of APX contained enzyme extract (50 μL), ascorbic acid (0.5 mM), sodium phosphate buffer (50 mM, pH 7.0), H_2_O_2_ (1 mM), EDTA (0.2 mM. The reaction was measured at 290 nm over 3 min. APX activity was calculated using molar extinction coefficient ε = 2.8 mM^−1^ cm^−1^ [45]. The protein content of the extracts was assayed using bovine serum albumin [46].

#### 2.3.5 Hydrogen peroxide concentration

200 mg of fresh leaves (30 days after flowering) were powdered with trichloroacetic acid (5 ml of 0.1% (w/v)) in an ice bath. After centrifuging (13000 rpm for 20 min), phosphate buffer (0.5 mL of 5 Ml, pH 7.0) and potassium iodine (1 mL of 1 mM) was added along with 0.2 mL supernatant. The absorbance was read at 390 nm. Hydrogen peroxide was measured using a standard curve of H_2_O_2_ [47].

#### 2.3.6 Malondialdehyde concentration

Briefly, 200 mg of fresh leaves (30 days after flowering) were milled with 5 mL of 10% thiobarbituric acid (TBA) and centrifuged (16000 rpm for 20 min). Then, 1.0 mL of the supernatant was added to 1 mL of 0.5% TBA, heated at 100 °C for 15 min, and cooled in an ice bath. The absorbance was obtained at 532 nm and 600 nm. Malondialdehyde concentration was measured using the extinction coefficient of malondialdehyde at 532 nm (155 mM^1^ cm^−1^) [48].

### 2.4 Seed quality

#### 2.4.1 Seed weight

The number of achenes per plant and the number of seeds per plant were measured. To obtain the mean seed weight, a batch of dry seeds was weighed and divided by the number of weighed seeds [49].

#### 2.4.2 Seed germination

Seed germination was evaluated according to the standards of the International Seed Testing Association [50]. After that, in each Petri dish, 50 seeds were placed on Whatman filter paper, and 10 ml of water was added to each Petri dish. A germination test was performed at 10 °C, 16/8 h (light/darkness), and 85% RH for 14 days. Seeds germinated were counted every day; afterward, the seed germination potential, mean germination time (MGT), and vigor index [51] were calculated according to the following equations [[3], [4], [5]]:

[3] Germination (%)=(germinated seeds number at initial stage(the fifth day)/total saples number) × 100

[4] MGT = Σ(Dn)/Σn

Where n is the number of germinated seeds on day D, and D is the number of days from the beginning of the germination test.

[5] Vigor Index (VI) = Gp (Germination percentage) × SL

In vigor index, Gp; germination percentage, and SL; shoot length of seedling.

#### 2.4.3 Seed longevity

The test of seed longevity was done by incubating seeds at 40 °C with 100% relative humidity in a closed tank with circulation for one day [50]. The seeds were then taken out, and their germination was counted.

### 2.5 Seed chemical compositions

#### 2.5.1 Seed oil and fatty acid components

Dry seeds (1 g) were milled and wrapped in a thimble of extraction paper. Papers were placed inside the distillation flask in a soxhlet, and hexane solution (150 mL) was added. The solvent was placed at a range of boiling temperatures (40–60 °C) for 4 h to evaporate. The oil extraction was obtained by filtration and dehydration [52]. Eventually, the relative percentage of fatty acids was measured by a gas chromatograph system [53].

#### 2.5.2 Seed mucilage

Dried seeds (10 g) were boiled at 100 °C for 30 min. After adding ethanol 80% (v/v) to the reaction, the samples were centrifuged at 30000 rpm for 40 min [54].

#### 2.5.3 Seed sucrose

Dry seeds (0.3 g) were mixed with 80% ethanol (5 mL). The reaction was heated at 80 °C for 40 min and centrifuged at 100000 rpm for 10 min. The sucrose was estimated with a mixture of 0.15 mL supernatant and NaOH (0.15 mL 2 mM) at 100 °C for 5 min, which was then mixed together with HCL (2.1 mL 30%) and resorcinol (0.6 mL 0.1%) at 80 °C for 10 min. The reaction was read at 480 nm using sucrose as one standard [55].

### 2.6 Statistical analysis

The data were analyzed using SAS (SAS version 9.2, SAS Institute, Cary, NC, USA). Duncan's multiple range test (0.05%) was done to make a mean difference comparison. Origin Pro Software (OriginLab Corporation, Northampton, MA, USA) was used to run the Pearson correlation.

## 3 Results

An increase in chlorophyll *a* and *b* concentration was obtained in mother plants grown under standard temperature (25 °C). Furthermore, chlorophyll *a* and *b* concentrations increased in mother plants that were grown under the long day (16 h d^-1^). In comparison with *L. royleana*, a rise in chlorophyll *a* and *b* concentrations was observed in *L. iberica* leaves (Table 4).

**Table 4:** Means comparison of physiological and biochemical indices, number achenes per plant, number seeds per plan and seed weight of Lallemantia species under different temperature and photoperiod treatments.

Plant species	Temperature (°C)	Photoperiod (hd^−1^)													
			Chl a (μg.g^−1^)	Chl b (μg.g^−1^)	OS (%)	CS (%)	TSS (mg.g^−1^)	SOD (Units/mg protein)	CAT (Units/mg protein)	APX (Units/mg protein)	H_2_O_2_ (nmol. g^−1^)	MDA (nmol. g^−1^)	NAPP	NSPP	SW (g)
*L.iberica*	T1	P1	0.34 ± 0.001d	0.2 ± 0.001d.f	68.33 ± 0.88e	59 ± 0.58ef	35.63 ± 0.45i	5.4 ± 0.02g	4.09 ± 0.01h	1.76 ± 0.01l	61.43 ± 0.48c	46.35 ± 0.08d	462.67 ± 1.45c	13453 ± 0.93d	0.002 ± 0.0001e
		P2	0.36 ± 0.003c	0.21 ± 0.001b	72.67 ± 0.67c	42 ± 0.58j	38.73 ± 0.28g	6.4 ± 0.09f	4.15 ± 0.01h	2.1 ± 0.01j	59.99 ± 0.28de	43.63 ± 0.03f	471.67 ± 1.45 ab	13624 ± 135.03c	0.002 ± 0.0001 fg
		P3	0.32 ± 0.002ef	0.2 ± 0.001ef	64.67 ± 0.33 fg	64 ± 0.58d	33.27 ± 0.07j	4.38 ± 0.11h	3.77 ± 0.06j	1.42 ± 0.01o	63.58 ± 0.15b	47.81 ± 0.4c	452.33 ± 1.2d	13213 ± 150.51g	0.003 ± 0.0001b
	T2	P1	0.37 ± 0.003b	0.21 ± 0.001b	75 ± 0.58b	55 ± 0.58g	37.42 ± 0.12h	6.58 ± 0.1f	4.17 ± 0.01h	1.89 ± 0.01k	58.13 ± 0.41f	42.34 ± 0.13g	475.67 ± 2.19a	13754 ± 8.19b	0.002±0cd
		P2	0.38 ± 0.004a	0.23 ± 0.001a	88 ± 0.58a	32.67 ± 1.45l	41.47 ± 0.14de	8.8 ± 0.28c	4.21 ± 0.01gh	2.32 ± 0.04i	53.72 ± 0.71h	41.44 ± 0.06h	474.67 ± 9.35a	14000 ± 5.7a	0.002 ± 0.0001 fg
		P3	0.36 ± 0.002c	0.2 ± 0.003cd	70.67 ± 0.67d	59.33 ± 0.88e	35.59 ± 0.1i	5.51 ± 0.08g	4.11 ± 0.01h	1.64 ± 0.01 m	60.23 ± 0.24de	44.39 ± 0.11e	462.67 ± 1.2c	13354.93 ± 6.29e	0.001 ± 0.0001hi
	T3	P1	0.32 ± 0.002 fg	0.18 ± 0.002g	63.33 ± 0.88 fg	68.33 ± 0.88c	31.38 ± 0.09k	4.44 ± 0.12h	3.76 ± 0.06j	1.52 ± 0.01n	63.18 ± 0.43b	49.66 ± 0.01b	444.67 ± 0.88de	13319 ± 9.5f	0.002 ± 0.0001cd
		P2	0.34 ± 0.003d	0.2 ± 0.001ef	68 ± 0.58e	48 ± 1.15i	34.36 ± 0.09j	5.29 ± 0.07g	3.9 ± 0.02i	1.76 ± 0.01l	60.79 ± 0.23cd	47.47 ± 0.08c	465.67 ± 1.76bc	13765 ± 11.85b	0.002 ± 0.0001ef
		P3	0.31 ± 0.001h	0.17 ± 0.002h	55 ± 0.58j	87.33 ± 0.88a	29.5 ± 0.03l	3.25 ± 0.1i	3.65 ± 0.02j	1.31 ± 0.01p	65.12 ± 0.28a	51.57 ± 0.92a	437 ± 1.53e	13090 ± 1.76h	0.004 ± 0.0002a
*L.royleana*	T1	P1	0.32 ± 0.003 fg	0.19 ± 0.001g	65 ± 0.58f	55 ± 0.58g	46.47 ± 0.61c	7.52 ± 0.09e	4.81 ± 0.05d	3.73 ± 0.01e	56.39 ± 0.08g	41.45 ± 0.1h	322±1h	11265 ± 3.65 m	0.002±0 fg
		P2	0.33 ± 0.001e	0.19 ± 0.002f	68.67 ± 0.88e	38.33 ± 0.88k	50.37 ± 0.49b	9.43 ± 0.06b	5.12 ± 0.01c	4.51 ± 0.01b	53.51 ± 0.15h	40.25 ± 0.13i	334.33 ± 0.33g	11703 ± 14.19j	0.003±0b
		P3	0.27 ± 0.003i	0.17 ± 0.001h	60.33 ± 0.33i	60 ± 0.58e	40.73 ± 0.27ef	6.44 ± 0.06f	4.53 ± 0.09f	3.27 ± 0.01h	57.4 ± 0.13 fg	43.22 ± 0.07f	313.67 ± 0.88ij	11108.33 ± 2.33o	0.002±0cd
	T2	P1	0.33 ± 0.001e	0.21 ± 0.003c	67 ± 1.15e	51 ± 0.58h	49.48 ± 0.55b	9.37 ± 0.09b	5.26 ± 0.03b	3.86 ± 0.01d	52.45 ± 0.06i	38.31 ± 0.12j	330.67 ± 1.76g	11400 ± 1.53l	0.002±0 fg
		P2	0.34 ± 0.002d	0.22 ± 0.002b	76 ± 0.58b	34.33 ± 0.33l	57.9 ± 0.91a	12.42 ± 0.07a	5.53 ± 0.05a	4.89 ± 0.01a	49.9 ± 0.7j	33.46 ± 0.04k	342.67 ± 1.2f	11890 ± 17.42i	0.001±0hi
		P3	0.32 ± 0.003g	0.2 ± 0.001de	62.67 ± 0.33gh	56.67 ± 0.67 fg	45.47 ± 0.64c	8.47 ± 0.07d	5.02 ± 0.01c	3.55 ± 0.01f	54.41 ± 0.09h	40.28 ± 0.13i	321.67 ± 1.2hi	11122.33 ± 1.2n	0.002±0cd
	T3	P1	0.25 ± 0.004j	0.17 ± 0.002h	61 ± 0.58hi	62.67 ± 1.45d	42.09 ± 0.46d	6.55 ± 0.13f	4.63 ± 0.07ef	3.44 ± 0.01g	59.42 ± 0.11e	44.54 ± 0.17e	310.33 ± 0.88j	11108.67 ± 3.18o	0.002±0ef
		P2	0.28 ± 0.001i	0.19 ± 0.002g	64.33 ± 0.33 fg	44 ± 0.58j	46.07 ± 0.34c	7.51 ± 0.07e	4.73 ± 0.03de	4.12 ± 0.01c	57.04 ± 0.32g	43.49 ± 0.13f	315 ± 0.58hij	11500 ± 17.19k	0.004±0a
		P3	0.23 ± 0.001k	0.16 ± 0.001i	44.67 ± 0.88k	77 ± 0.58b	39.8 ± 0.25 fg	5.62 ± 0.1g	4.3 ± 0.02g	3.24 ± 0.05h	61.76 ± 0.56c	47.57 ± 0.22c	279.67 ± 3.84k	10589.87 ± 301.15p	0.003±0b

The same letters in each column show non-significant differences at p < 0.05, analyzed by Duncan's multiple range test. The same letters in each column show non-significant differences at p < 0.05, analyzed by Duncan's multiple range test. T1: low temperature (15 °C); T2: standard temperature (25 °C); T3: high temperature (35 °C); P1: short day (8 hd-1), P2: long day (16 hd-1); P3: Continuous light (24 hd-1); Chl a: chlorophyll *a*; Chl b: chlorophyll *b*; OP: open stomata; CS: closed stomata; TSS: total soluble sugar; SOD: superoxide dismutase; CAT: catalase enzyme activity; APX: ascorbate peroxidase; MDA: malondialdehyde; H2O2: hydrogen peroxide NAPP: number achenes per plant; NSPP: number seeds per plant; SW: seed weight.

The most open stomata and the least closed stomata were obtained in maternal plants grown under standard temperature (25 °C). In addition, an increase in open stomata and a decline in closed stomata in both species were obtained in mother plants matured under long day (16 h d^-1^). It is evident that *L. iberica* had the greatest percentage of open stomata and the least percentage of closed stomata in comparison with *L. royleana* (Table 4).

The otal soluble sugar content measured in both species' leaves were affected by temperature regimes and photoperiod. A decline in total soluble sugar content was observed in mother plants which were grown under high temperature (35 °C) and continuous light (24 h d^-1^). The total soluble sugar in the leaves of *L. royleana* was higher than that in *L. iberica* (Table 4).

The SOD, CAT, and APX enzyme activities in both species of *Lallemantia* significantly increased as the temperature increased from 15 °C to 25 °C. There was an upward trend in SOD, CAT, and APX enzyme activities in maternal plants grown under long day (16 h d^-1^). Compared with *L.* *iberica*, the highest SOD, CAT, and APX enzyme activities were observed in *L. royleana* (Table 4).

Maternal temperature treatments significantly influenced H_2_O_2_ and MDA concentrations, such that H_2_O_2_ and MDA were enhanced by increasing temperature (35 °C). Increases in H_2_O_2_ and MDA concentrations were obtained in the leaves of mother plants grown under continuous light (24 h d^-1^). There was a decline in H_2_O_2_ and MDA concentrations in the leaves of *L. royleana* in comparison with *L. iberica* (Table 4).

The most achenes, seeds per plant, and seed weight were observed in mother plants grown under high temperatures (25 °C) and long days (16 h d^-1^). Compared with L. *royleana*, the greatest achenes per plant, seeds per plant, and seed weight were observed in *L. iberica* (Table 4).

A rise in germination and vigor index were observed in seeds matured under high temperature (25 °C). The germination and vigor index were higher in seeds matured under long days (16 h d^-1^) than in other levels of photoperiod. The germination and vigor index in L. iberica were higher than those in *L. royleana.* MGT was found to be lowest in seeds matured at standard temperature (25 °C) and long day (16 h d^-1^). MGT in *L. royleana* seeds was lower compared with *L. iberica* (Table 5).

**Table 5:** Means comparison of germination indices, seed logevity, fatty acids content, seed oil, mucilage and sucrose content of *Lallemantia* species under different temperature and photoperiod treatments.

Plant species	Temperature (°C)	Photoperiod (hd-1)										
			GP (%)	MGT (h)	VI	SL (%)	Linolenic Acid (%)	Linoleic acid (%)	Oleic Acid (%)	Seed oil (%)	Seed mucilage (%)	Seed Sucrose (%)
*L.iberica*	T1	P1	90.77 ± 0.27f	23.3 ± 0.06i	776.61 ± 2.05e	74.13 ± 0.37f	9.44 ± 0.12d	51.45 ± 0.54e	13.29 ± 0.08f	28.84 ± 0.41c	7.44 ± 0.13h.j	45.35 ± 0.07i
		P2	93 ± 0.35cd	19.53 ± 0.03 m	863.12 ± 10.75d	77.93 ± 0.62e	11.4 ± 0.09b	55.47 ± 0.64c	16.53 ± 0.13b	30.4 ± 0.09b	8.48 ± 0.15g	47.55 ± 0.1h
		P3	87.3 ± 0.3i	25.37 ± 0.03g	644.04 ± 14.07i	68.86 ± 0.46g	7.42 ± 0.09f	47.2 ± 1.92ghi	11.47 ± 0.09h	27.14 ± 0.31d	7.11 ± 0.01j	43.47 ± 0.09j
	T2	P1	93.33 ± 0.58c	21.3 ± 0.06k	874.84 ± 11.49d	82.43 ± 0.7e	10.78 ± 0.23c	58.08 ± 0.25b	15.07 ± 0.33d	30.44 ± 0.1b	7.96 ± 0.04h	48.46 ± 0.04h
		P2	99.67 ± 0.33a	16.47 ± 0.03n	1031.71 ± 19.02b	81.4 ± 0.52bc	13.44 ± 0.13a	66.45 ± 0.05a	18.81 ± 0.03a	32.44 ± 0.08a	9.23 ± 0.06f	57.51 ± 0.05e
		P3	91.6 ± 0.35ef	22.27 ± 0.03j	775.09 ± 6.85ef	71.4 ± 0.3f	8.37 ± 0.12e	52.16 ± 0.83de	13.27 ± 0.07f	28.98 ± 0.33c	7.34 ± 0.06ij	44.46 ± 0.07i
	T3	P1	88.4 ± 0.4gh	26.87 ± 0.37f	661.01 ± 8.02hi	71.46 ± 0.88h	7.36 ± 0.07f	50.52 ± 0.5ef	11.36 ± 0.04h	27.88 ± 0.23d	7.19 ± 0.03j	40.31 ± 0.11k
		P2	90.73 ± 0.24f	21.37 ± 0.03k	740.18 ± 15.99e g	74 ± 0.49g	9.41 ± 0.13d	51.8 ± 0.36de	14.34 ± 0.08e	30.13 ± 0.3b	7.87 ± 0.26hi	44.61 ± 0.08i
		P3	82.83 ± 0.27k	30.43 ± 0.12c	540.94 ± 24.48j	69.87 ± 0.76h	6.44 ± 0.06g	44.91 ± 0.3jk	9.5 ± 0.09i	25.15 ± 0.44f	6.84 ± 0.03j	37.23 ± 0.86l
*L.royleana*	T1	P1	88.87 ± 0.32g	28.6 ± 0.12d	659.11 ± 7.37hi	80.7 ± 0.25cd	7.51 ± 0.09f	47.9 ± 0.91gh	11.33 ± 0.06h	25.11 ± 0.14f	16.3 ± 0.46d	60.35 ± 0.07c
		P2	91.1 ± 0.28ef	24.47 ± 0.09h	944.53 ± 13.84c	82.43 ± 0.07b	9.41 ± 0.13d	51.17 ± 0.3e	14.38 ± 0.11e	27.52 ± 0.49d	17.97 ± 0.09b	62.55 ± 0.1b
		P3	84.82 ± 0.4j	30.47 ± 0.03c	556.48 ± 15.43j	79.37 ± 0.09d	6.37 ± 0.09g	45.03 ± 0.27i.k	9.55 ± 0.1i	23.78 ± 0.27g	16.23 ± 0.45d	58.47 ± 0.09d
	T2	P1	91.98 ± 0.39de	25.33 ± 0.03g	864.71 ± 7.67d	82.44 ± 0.23b	9.4 ± 0.09d	53.78 ± 0.36cd	13.54 ± 0.08f	27.27 ± 0.18d	18.07 ± 0.09b	63.46 ± 0.04b
		P2	98.23 ± 0.32b	20.4 ± 0.06l	1081.48 ± 10.74a	85.19 ± 0.66a	11.37 ± 0.14b	58.32 ± 0.36b	15.9 ± 0.32c	30.27 ± 0.13b	23.51 ± 0.06a	73.44 ± 0.98a
		P3	90.71 ± 0.25f	27.5 ± 0.06e	683.19 ± 6.98h	80.89 ± 0.26cd	6.4 ± 0.09g	48.93 ± 0.36 fg	11.35 ± 0.03h	26.3 ± 0.24e	17.01 ± 0.05c	59.43 ± 0.07cd
	T3	P1	85.83 ± 0.34j	31.53 ± 0.09b	631.95 ± 6.95i	79.46 ± 0.04d	5.36 ± 0.07h	44.33 ± 0.55k	9.44 ± 0.1i	24.27 ± 0.15g	16.99 ± 0.07c	55.67 ± 0.27f
		P2	87.39 ± 0.09hi	26.33 ± 0.09f	728.13 ± 8.77g	80.72 ± 0.29c	8.33 ± 0.16e	46.58 ± 0.58hij	12.25 ± 0.07g	25.33 ± 0.21f	17.57 ± 0.03b	58.53 ± 0.19d
		P3	74.5 ± 0.64l	35.17 ± 0.68a	500 ± 5.65k	77.91 ± 0.32e	4.21 ± 0.06i	38.22 ± 1.31l	8.25 ± 0.1j	23.41 ± 0.03g	15.56 ± 0.04e	53.37 ± 0.09g

The same letters in each column show non-significant differences at p < 0.05, analyzed by Duncan's multiple range test. The same letters in each column show non-significant differences at p < 0.05, analyzed by Duncan's multiple range test. T1: low temperature (15 °C); T2: standard temperature (25 °C); T3: high temperature (35 °C); P1: short day (8 hd-1), P2: long day (16 hd-1); P3: Continuous light (24 hd-1); GP: germination percentage; MGT: mean germination time, VI: vigor index; SL: seed longevity.

A reduction in longevity was indicated in seeds matured under high temperature (35 °C). However, the longevity of seeds matured under long day (16 h d^-1^) was more than that of other photoperiod treatments. Based on the results, an increase in longevity of L. royleana seeds was observed compared with *L. iberica* seeds (Table 5).

Seeds matured under standard temperature (25 °C) had a higher oil content and relative percentage of fatty acids. Furthermore, as day length increased, the oil content and fatty acids of LNA, LA, and OA in seeds matured decreased. Oil content and relative percentages of LNA, LA, and OA were higher in mature *L. iberica* seeds than in *L. royleana* seeds (Table 5). (Table 5)*.*

Standard temperature (25 °C) had a significant effect on the increase in mucilage content of seeds compared with other temperature regimes. Results demonstrated that mucilage content increased from short day (8 h d^-1^) to long day (16 h d^-1^) in mature seeds. Compared with *L.* *iberica* seeds, the highest mucilage was obtained in *L. royleana* seeds (Table 5).

The highest and lowest sucrose contents were observed in seeds matured under standard (25 °C) and high (35 °C) temperatures. Furthermore, seeds matured under long day (16 h d^-1^) conditions had higher sucrose content than those other photoperiod treatments. Compared with *L. iberice* seeds, the of seeds *L. royleana* had the greatest sucrose content (Table 5).

**Fig. 2:**
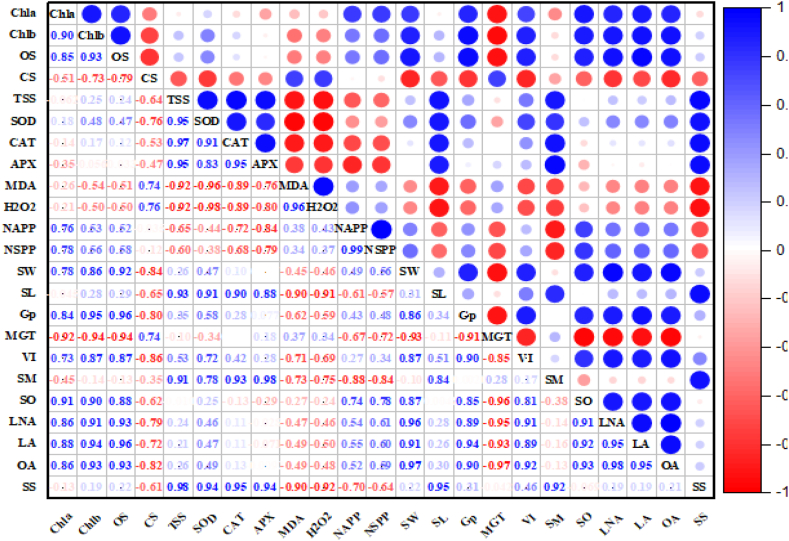
Pearson correlation among studied traits in both *Lallemantia* species Chla: chlorophyll *a*; Chlb: chlorophyll *b*; OP: open stomata; CS: closed stomata; TSS: total soluble sugar; SOD: superoxide dismutase; CAT: catalase enzyme activity; APX: ascorbate peroxidase MDA: malondialdehyde; H_2_O_2_: hydrogen peroxide; NAPP: number achenes per plant; NSPP: number seeds per plant; SW: seed weight; GP: germination percentage; MGT: mean germination time, VI: vigor index; SL: seed longevity; SM: seed mucilage; SO: seed oil; LNA: linolenic acid; LA: linoleic acid; OA: oleic acid; SS: seed sucrose.

The values of the Pearson correlation showed that seed weight had a positive correlation with open stomata, chlorophyll *a*, chlorophyll *b*, germination, and vigor index. It was clear that seed oil and seed mucilage had a positive correlation with seed weight, chlorophyll *a*, chlorophyll *b*, and open stomata. Besides, there was a positive correlation between seed longevity, seed mucilage, and seed sucrose content. Total soluble sugar, superoxidase dismutase, catalase, and ascorbate peroxidase activities were negatively correlated with MDA and H_2_O_2_. In addition, there was a negative correlation between MGT and germination, vigor index, and seed weight (Fig. 2).

## 4 Discussion

The maternal plant's environment during seed filling strongly influences seed quality, physiological and biochemical properties, and indices of seed quality [1,56]. An important photosynthetic pigment of the maternal plant is chlorophyll which determines the capacity for photosynthetic activity and the growth of the maternal plant [14]. The results suggested that standard temperature (25 °C) and long day (8 h d^-1^) conditions of the maternal plant's environment induced an increase in chlorophyll concentrations of both *Lallemantia* species which is most probably due to an increase in open stoma stomata [57]. An increase in open stomata allows for the intake of CO_2_ which is required for the process of photosynthesis [58]. Therefore, an increase in CO_2_ availability increases photosynthesis and drops the oxygenase activity of Rubisco [59]. Some studies have demonstrated that open and closed stomata on the leaves of the mother plant play a vital role in gas exchange, transpiration, and photosynthesis [14]. Higher chlorophyll concentration *in L. iberica* leaves than that in *L. royleana* can be due to an increase in open stomata [36].

Soluble sugar content could be a regulator in defense mechanisms, inducing the balancing of ROS in cells under different regimes of temperature and light [60]. This soluble sugar content acts as a scavenger of ROS and a membrane stabilizer against stress [61]. In this study, total soluble sugar content in the maternal plant leaves increased as it matured under standard temperature (25 °C) and long day (16 h d^-1^). The accumulation of soluble sugar in the mother plant's leaves appears to be caused by osmotic stress which is exacerbated by the high temperature and light of the maternal plant's environment [62]. Some studies have indicated that sugar acts as an antioxidant and causes the improvement of oxidative stress [63]. In comparison with *L. iberica*, further total soluble sugar in *L. royleana* can probably be attributed to the greater stability of the membrane against ROS [60].

Based on biochemical and molecular findings, it has been determined that decrease in membrane structure destruction prevents the production and accumulation of MDA and H_2_O_2_ [64]. The reduction in MDA and H_2_O_2_ concentration under standard temperature (25 °C) and long day (16 h d^-1^) can be associated with increasing SOD, CAT, and APX enzyme activities which act as the main line of defense [65]. It seems that the reduction in lipid peroxidation under standard temperature and long day during seed maturation indicates the reduction in membrane damage, and provides the essential carbon for the maintenance of raffinose and polysaccharides in the offspring [66]. According to recent research, sucrose, and raffinose are involved in the maintenance of cytoplasm glassy state and reduce lipid peroxidation [30,67]. In the current study, more SOD, CAT, and APX enzyme activities in *L. royleana* than that in *L. iberica* leaves were probably due to the limitated oil content, and reduction in the accumulation of H_2_O_2_ and lipid peroxidation [64,68].

In our study, growth traits such as achenes per plant and seeds per plant, and seed weight decreased under high temperature (35 °C) and continuous light (24 h d^-1^), which can be attributed to a decline in chlorophyll concentrations and uptake of CO_2_ availability for photosynthesis [69]. Numerous studies have shown a rise in the uptake of CO_2_ during photosynthesis stimulates carbohydrates accumulation in leaves [38,70]. The transportation of carbohydrates from the source (leaf) to the sink (seed) has a crucial pattern in the growth and development of seeds [71]. On the other hand, it is probably the transgenerational epigenetic inheritance that supplies the mechanisms for the adaptation of maternal effects on offspring phenotypes such as seed weight, germination, seedling growth, and seed composition [72,73]. Our results showed that achenes per plant and seeds per plant, and seed weight were more in *L. iberica* than that in *L. royleana*, which can be attributed to the much higher seed weight of *L. iberica* [74,75].

An increase in germination and vigor index and decrease in MGT have been observed in some plants such as Brassica napus L. [76], and Orize sativa L [77]. under standard temperature and a long day. It has been reported that the increase in photosynthetic activity in the leaves of maternal plants can induce the production and accumulation of carbohydrates inside seeds [78]. In fact, carbohydrates are a crucial source of cell energy and assist the seeds in germination [79]. Starch is a strong carbohydrate that provides essential energy for the hydrolysis action of a and b amylase for the germination of aged seeds [80]. A rise in the germination and vigor index of *L. iberica* seeds compared with *L. royleana* seeds can be attributed to the increased seed weight [81]. On the other hand, the higher MGT of *L. royleana* seeds was probably due to the high mucilage around the seeds [40]. In other words, the presence of mucilage around the seed serves as a filter or sorbent under stress conditions and creates a hydrophilic property [82].

An increase in longevity of developed seeds under standard temperature (25 °C) and long day conditions can be attributed to high photosynthetic pigments of maternal plants which supply more carbohydrates for synthesizing starch, lipids, and proteins in seeds [83]. Some studies documented that carbohydrates, as the main reserves of seeds, induced the glassy state of the cell cytoplasm and inhibited the deterioration of seeds [84]. The glassy state raises the viscosity of the intracellular fluid and decreases metabolic activity in the cytoplasm [85]. Further seed longevity of *L. royleana* than that in *L. iberica* may be due to less oil in *L. royleana* seeds [86]. Hence, it can be inferred, high saturated fatty acids during seed storage induce a decline in the lipid fluidity of cell membranes and a rise in MDA and ROS accumulation [87]. In addition, the decline in the longevity of *L. iberica* may be due to the increase in oxidative damage in polyunsaturated lipids [88,89].

The highest oil content, relative percentage of fatty acids, and mucilage content were found in seeds that were matured under standard temperature (25 °C) and long day (16 hd-1). The reason could be attributed to increased chlorophyll content [90,91]. Obviously, photosynthesis activities in the leaves play an important role in the generation and transportation of carbohydrates into seeds in order to Ref. [92] produce the storage reserves such as lipid, protein, and starches [93]. The higher oil content and relative percentage of fatty acids *in L. iberica* seeds than in *L. royleana* seeds could be attributed to an increase in photosynthetic pigments and open stomata, which provide more carbohydrates for the biosynthesis of mucilage, oil, and fatty acids [38]. Furthermore, the increase in the oil and fatty acids of *L. iberica* seeds may be related to higher levels of nutrients such as nitrogen, phosphorus, potassium, calcium, and manganese [94,95]. Some studies have indicated that phosphorus in seeds has an important role in providing ATP and NADPH for biosynthetic fatty acids [38]. The results showed an increase in mucilage of *L. royleana* seeds in comparison with *L. iberica*, which may be due to epigenetic memory superseding that of maternal plants [23].

The reduction in sucrose content under standard temperature (25 °C) and long day (16 h d^-1^) conditions may be related to an rise in chlorophyll content [96]. Chlorophyll is a crucial pigment that plays a role in the absorption, transmission, and transformation of light energy in photosynthesis [97]. The production of photosynthetic activity such as sucrose occurs in the carbon vertebrae, which provide energy for the metabolism and synthesis of amino acids in seeds [98]. Some studies have been indicated that maternal plants assimilate inorganic carbon via photosynthetic reactions in the forms of sucrose, starch, glucose, and fructose [99]. The carbon absorbed by mother plant is transferred to the offspring in the form of starch and a fraction of sugars [100]. Compared with *L. iberica* seeds, an upward trend in sucrose content of *L. royleana* seeds was observed, which can be related to the genetic differences between maternal plants [101].

## 5 Conclusion

The findings of this study revealed that the temperature and light of the maternal plant's environment had a strong influence not only on the physio-biochemical properties of maternal plants but also on the seed quality and chemical compositions of their offspring. The appropriate response of *L. iberica* and *L. royleana* maternal plants to the standard temperature (25 °C) and long day (16 h d^-1^) environment improved offspring weight, germination, vigor index, longevity, mucilage, oil, the relative percentage of fatty acids, and sucrose content. Furthermore, due to the higher dependency of *L. iberica* maternal plant on the temperature and light of the maternal plant's environment than *L. royleana*, the highest weight, germination, vigor, oil, and relative percentage of fatty acids were observed in the offspring of *L. iberica.* On the other hand, further longevity, mucilage, and sucrose observed in offspring of *L. royleana* compared with *L. iberica* was probably due to genetic reasons. Generally, this research highlighted how temperature and light in the environment could affect the maternal plants and offspring of *L. iberica* and *L. royleana.* However, it is more necessary to investigate the epigenetic mechanisms of maternal plants on the offspring of *L. iberica* and *L. royleana* in future research.

## Author contribution statement

Arezoo Paravar: Conceived and designed the experiments; Performed the experiments; Analyzed and interpreted the data; Contributed reagents, materials, analysis tools or data; Wrote the paper.

Saeideh Maleki Farahani: Conceived and designed the experiments; Interpreted the data Contributed reagents, materials and analysis data.

Alireza Rezazadeh: Contributed reagents, materials, analysis tools or data.

## Data availability statement

Data included in article/supplementary material/referenced in article.

## Additional information

No additional information is available for this paper.

## Abbreviations

T1: low temperature (15 °C)
T2: standard temperature (25 °C)
T3: high temperature (35 °C)
P1: short day (8 h d^-1^)
P2: long day (16 h d^-1^)
P3: Continuous light (24 h d^-1^)
R:B: ratio: ratio of red light to blue light
ROS: reactive oxygen species
Chl a: chlorophyll *a*
Chl b: chlorophyll *b*
OP: open stomata
CS: closed stomata
TSS: total soluble sugar
SOD: superoxide dismutase
CAT: catalase enzyme activity
APX: ascorbate peroxidase
MDA: malondialdehyde
H_2_O_2_: hydrogen peroxide
SW: seed weight
GP: germination percentage
MGT: mean germination time
VI: vigor index
SL: seed longevity
SM: seed mucilage
SO: seed oil
LNA: linolenic acid
LA: linoleic acid
OA: oleic acid
SS: seed sucrose
